# Core-Fucosylated Tetra-Antennary *N*-Glycan Containing A Single *N*-Acetyllactosamine Branch Is Associated with Poor Survival Outcome in Breast Cancer

**DOI:** 10.3390/ijms20102528

**Published:** 2019-05-23

**Authors:** Harmin Herrera, Tinslee Dilday, Allison Uber, Danielle Scott, Joelle N. Zambrano, Mengjun Wang, Peggi M. Angel, Anand S. Mehta, Richard R. Drake, Elizabeth G. Hill, Elizabeth S. Yeh

**Affiliations:** 1 Graduate School of Biomedical Sciences and Professional Studies, Microbiology and Immunology Graduate Program, Drexel University College of Medicine, Philadelphia, PA 19129, USA; hh435@drexel.edu; 2 Department of Cell and Molecular Pharmacology and Experimental Therapeutics, Medical University of South Carolina, Charleston, SC 29425, USA; dilday@musc.edu (T.D.); scottd@musc.edu (D.S.); zambrano@musc.edu (J.N.Z.); wangm@musc.edu (M.W.); angelp@musc.edu (P.M.A.); mehtaa@musc.edu (A.S.M.); draker@musc.edu (R.R.D.); 3 Department of Pediatrics, Division of Hematology/Oncology, Medical University of South Carolina, Charleston, SC 29425, USA; uber@musc.edu; 4 Department of Public Health Sciences, Medical University of South Carolina, Charleston, SC 29425, USA; hille@musc.edu

**Keywords:** breast cancer, *N*-glycan, core-fucose, *N*-acetyllactosamine

## Abstract

(1) Glycoproteins account for ~80% of proteins located at the cell surface and in the extracellular matrix. A growing body of evidence indicates that α-L-fucose protein modifications contribute to breast cancer progression and metastatic disease. (2) Using a combination of techniques, including matrix-assisted laser desorption/ionization imaging mass spectrometry (MALDI-IMS) based in cell and on tissue imaging and glycan sequencing using exoglycosidase analysis coupled to hydrophilic interaction ultra-high performance liquid chromatography (HILIC UPLC), we establish that a core-fucosylated tetra-antennary glycan containing a single *N*-acetyllactosamine (F(6)A4G4Lac1) is associated with poor clinical outcomes in breast cancer, including lymph node metastasis, recurrent disease, and reduced survival. (3) This study is the first to identify a single N-glycan, F(6)A4G4Lac1, as having a correlation with poor clinical outcomes in breast cancer.

## 1. Introduction

Fucosylation is a relatively well-defined biomarker for progression in many human cancers; for example, pancreatic and hepatocellular carcinoma [1,2,3,4,5,6,7,8], but its role in breast cancer is much less well defined. In mammals, fucose is exclusively used in the L-configuration (i.e., α-l-fucose). It is added to proteins in three major configurations: Core (an α-1,6-linkages to the core *N*-acetylglucosamine adjacent to asparagine), *O*-linked (on serine or threonine), and terminal/subterminal positions along the sugar chain. Thirteen major fucosylation related gene products have been identified. These are *FUT1-11, POFUT1*, and *POFUT2,* where *FUT1-7, 9-11* regulate terminal/subterminal (outer arm) fucosylation, *FUT8* regulates core fucosylation, and *POFUT1* and *POFUT2* regulate *O*-linked fucosylation. *FUT8, POFUT1*, and *POFUT2* are the only three genes that are required for development and genetic knockout models demonstrate that loss of each gene results in lethal abnormalities, including severe growth retardation and defects [9,10,11,12,13].

A growing body of evidence indicates that α-L-fucose protein modifications contribute to breast cancer progression and metastatic disease [14,15,16,17,18,19]. A genomic study that focused on a cohort of 194 breast carcinomas (Hamburg Cohort) reported on RNA expression data of 202 glycosylation genes generated by microarray analysis (Affymetrix HG-U133A) [20]. Although subtype data was lacking from this study, clear statistically significant correlations between *FUT1, FUT6, POFUT1*, as well as at least one fucose modifying enzyme, Fucosidase 1 (*FUCA1*), were shown to correlate with poor prognosis, with some having higher expression and some with lower expression in relation to breast cancer progression. Studies directly evaluating the contribution of *FUT8*, which regulates core fucosylation, to breast cancer progression are limited. However, studies show that *FUT8* expression in breast cancer patient samples correlated with lymph node metastasis, disease-free survival and overall survival [21,22].

Pharmacological inhibition of fucosylation suppresses mammary tumor cell migration and invasion [14,15,23]. Consistent with these observations, we have performed studies here to show that treatment of 4T1 metastatic mouse mammary tumor cells with another fucosylation processing inhibitor, the compound 2-deoxy-2-fluoro-l-fucose (2FF), which inhibits GDP-fucose synthesis, results in reduced cell migration using transwell migration assay. Furthermore in this study, we identify major core-fucosylated N-glycans in the metastatic 4T1 mammary tumor cell line model. Using a combination of techniques including matrix-assisted laser desorption/ionization imaging mass spectrometry (MALDI-IMS) based in cell and on tissue imaging and glycan sequencing using exoglycosidase analysis coupled to hydrophilic interaction ultra-high performance liquid chromatography (HILIC UPLC), we establish that a core-fucosylated tetra-antennary glycan containing a single *N*-acetyllactosamine branch is present in 4T1 cells, as well as human breast tumor tissues using tumor microarrays (TMA).

Additionally, analysis of two separate patient TMA cohorts demonstrates that this core-fucosylated tetra-antennary with single N-acetyllactosamine strongly correlates with poor patient outcomes. We found this *N*-glycan to be more highly expressed in tumor-bearing lymph nodes compared to normal lymph nodes, as well as tumor tissues from patients who had recurrences compared to those that did not. Furthermore, expression of this core-fucosylated tetra-antennary with one N-acetyllactosamine in early stage (stage 1 and 2) breast cancers was associated with reduced survival. Taken together, our findings are the first to demonstrate that a specific core-fucosylated N-glycan, containing a single N-acetyllactosamine branch, is strongly associated with poor survival in breast cancer.

## 2. Results

### 2.1. Fucosylation Regulates Cell Migration

**Figure 1:**
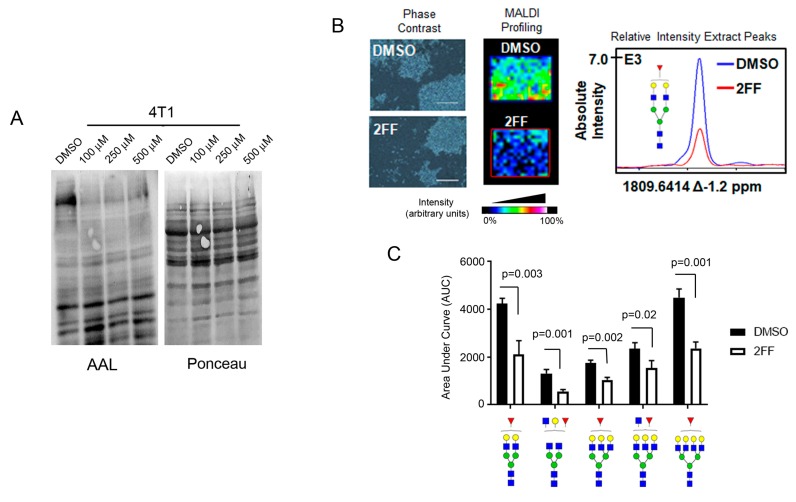
2-deoxy-2-fluoro-L-fucose (2FF) suppress fucosylation in 4T1 cells. (**A**) Decreased fucose detected by *Aleuria aurantia* lectin (AAL) blotting of 4T1 cell lysates treated with vehicle (DMSO) or increasing concentration (100–500 μM) of 2FF, a fucosylation inhibitor. Ponceau S stain was used to show total protein loading. (**B**) MALDI-IMS of 4T1 cells grown in tissue chambered slide and treated with DMSO or with 500 μM 2FF. Phase contrast (**left** panel) depicts cells from DMSO or 2FF chambers used in MALDI profiling experiments (**middle** panel). Scale bar = 400 µm The graph showing absolute intensity is shown (**right** panel). (**C**) The area under the curve (AUC) of normalized total ion count (TIC) was calculated and compared between DMSO control and 2FF treated samples. Glycan nomenclature: Blue square as GlcNAc, yellow circle as galactose, green circle as mannose, red triangle as fucose.

2FF inhibits de novo synthesis of GDP-fucose in mammalian cells [24]. To determine the effect of this compound on cell migration, we treated a mouse mammary tumor cell line representing a stage 4 metastatic breast cancer, 4T1, with 2FF. To show that 2FF was reducing fucosylation, we performed lectin blot using *Aleuria aurantia* lectin (AAL), a fucose-binding protein, on 4T1 cells that were treated with either DMSO (vehicle) or increasing concentrations of 2FF (100–500 μM). 2FF reduced AAL signal at all concentrations and increasing concentrations did not result in more profound inhibition of AAL signal (Figure 1A). To confirm that 2FF inhibited fucosylation, we performed matrix-assisted laser desorption ionization coupled to Fourier Transform Ion Cyclotron Resonance (MALDI FT-ICR) mass spectrometry on 4T1 cells that were either treated with DMSO or 2FF (Figure 1B). We observed a ~50% decrease in fucosylated forms of N-glycans detected (Figure 1C).

**Figure 2:**
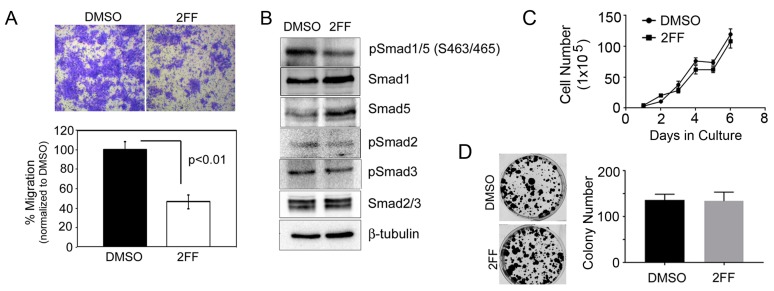
The role of fucosylation in regulating migration of 4T1 cells. (**A**) Crystal violet staining and quantitation of 4T1 cell migration. Cells were treated for 48-h with DMSO (control) or 100 μM 2FF prior to plating for the migration assay. Migration assays were imaged at 200× magnification. (**B**) 4T1 cells treated with DMSO or 100 μM 2FF were probed by Western blotting with antibodies against Smad2/3, and Smad1/5/8 signaling pathways. β-tubulin was used as loading control. (**C**) 1 × 10^5^ 4T1 cells were plated per well and counted each day for 7 days. Cells were treated with DMSO (control) or 100 μM 2FF on days 1, 3, and 5 of the assays. (**D**) 2 × 10^3^ 4T1 cells were plated per well and treated with DMSO or 100 μM 2FF. Media containing DMSO or 2FF was refreshed every three days. Cells were stained with crystal violet and counted on day 14. Colony formation was not affected by fucosylation in 4T1 cells.

To show that fucosylation is critical for mammary tumor cell migration, we treated 4T1 cells with 2FF for 48 h (hrs) and then seeded live cells for analysis by migration assay. 2FF treated cells were impaired in their ability to migrate compared to DMSO treated control cells (Figure 2A). To assess signaling molecules that regulate migration, we evaluated Smad proteins, which become activated in response to TGFβ. We found that 2FF decreased phosphorylation of Smad 1/5 and Smad 2 (Figure 2B). To determine if fucosylation is essential for 4T1 cell proliferation after DMSO or 2FF treatment, we evaluated cell doubling over time (Figure 2C) and colony forming capacity (Figure 2D) and found that 2FF did not affect cell proliferation by either assay.

### 2.2. Identification of Core-Fucosylated N-Glycans in 4T1 Cells

**Figure 3:**
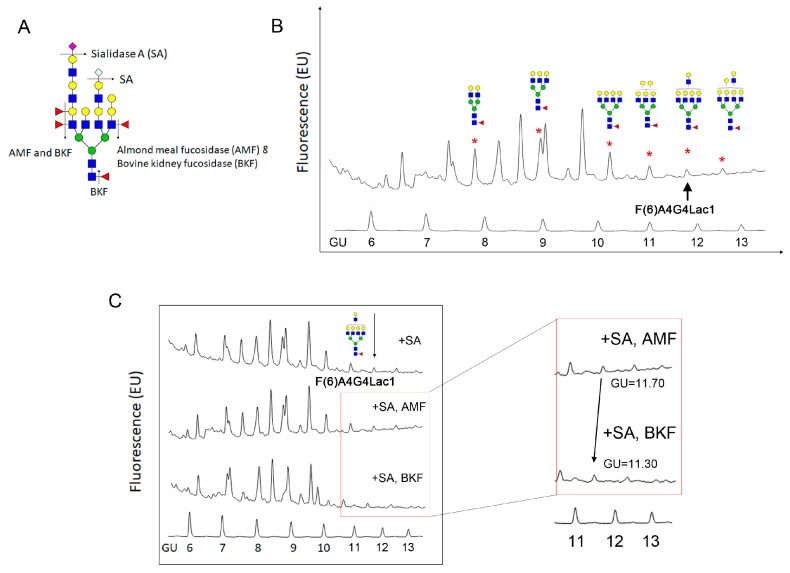
*N*-glycan sequencing of 4T1 cells. (**A**) Exoglycosidase enzymes used in this experiment. Sialidase A (SA) was used to remove mammalian N-acetyl neuraminic acid (NeuAc) or *N*-glycolylneuraminic acid (NeuGc); almond meal fucosidase (AMF) was used to remove outer arm α-1,2-, 1,3- and 1,4- link fucose; bovine kidney fucosidase (BKF) was used to remove all fucose residues including core α-1,6-linked fucose. (**B**) *N*-glycan chromatogram of 4T1 cells after being treated with SA and AMF. Several core-fucosylated glycans with different modifications (i.e., biantennary, tri-antennary, tetra-antennary, galactose, lactosamine) attributed to ~27.1% of total glycan in 4T1 cells. (**C**) Exoglycosidase digestions of 4T1 cells revealed ~1.5% of the total glycan was core fucosylated tetra-antennary glycan with one lactosamine arm/F(6)A4G4Lac1 (marked with an arrow). Insert, AMF and BKF treated samples shown following BKF digestion peak 11.70 glucose unit (GU) shifted to 11.30 GU confirmed the loss of one fucose residue at the α-1,6-linkage.

Prior studies suggest that core-fucosylation plays a critical role in breast cancer cell metastasis [21,23]. Therefore, to identify specific *N*-glycans with core-fucose that are found in 4T1 cells, we treated cells with Peptide: *N*-glycosidase F (PNGase F) to release *N*-glycans and then, with a sequential exoglycosidase digestion that included sialidase A (SA), almond meal fucosidase (AMF), which digests outer arm fucose and bovine kidney fucosidase (BKF), which digests all fucose including core fucose (Figure 3A). Following we labeled released sugars with 2-aminobenzamide (2AB) dye and separated individual glycans by HILIC UPLC. As a control for the AMF and BKF digestion, we treated a standard protein, ceruloplasmin, with each enzyme (Figure S1). Our analysis showed that at least six core-fucosylated *N*-glycans were present in 4T1 cells (Figure 3B). Core-fucosylation was confirmed by evaluating a shift in glucose units (GU) of individual glycans after BKF digestion. Shown here (Figure 3C), the desialylated core tetra-antennary glycan with a single *N*-acetyllactosamine, F(6)A4G4Lac1, which normally is seen at GU 11.70, showed no change in profile after AMF treatment but shifted to GU 11.30 after BKF treatment, indicating it is core-fucosylated.

### 2.3. Identification of Core-Fucosylated N-Glycans in 4T1-Derived Tumors

**Figure 4:**
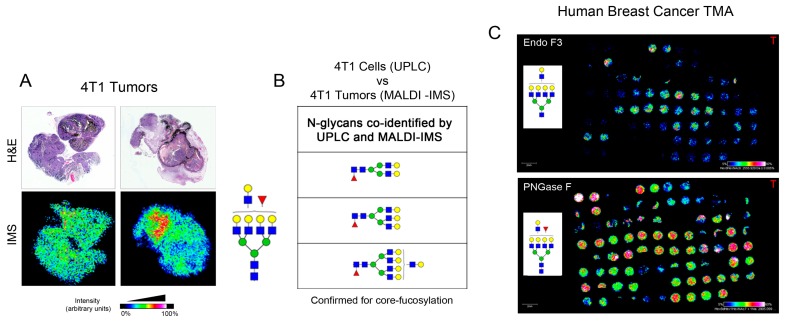
*N*-glycan MALDI-IMS of 4T1 tumor and human breast TMA. (**A**) A total of 1 × 10^5^ cells 4T1 injected into the abdominal mammary gland of BALB/c mice to generate tumors, which were collected five weeks post-injection. Collected tissues were FFPE blocked, sectioned, and prepared for MALDI-IMS. Spatial distribution of core fucosylated tetra-antennary with one lactosamine arm (*m*/*z* 2905.0359; A4G4F1Lac1) within the tumor is shown. H&E stain imaged at 10× magnification was performed to show the tumor region. (**B**) *N*-glycan found in MALDI-IMS and confirmed with UPLC for determination of core α-1,6-linked fucose. The *N*-glycans identified were core fucosylated bi-antennary, tri-antennary, and tetra-antennary with one lactosamine arm. (**C**) One panel representative of human breast TMAs from 33 total patients with clinical, tumor staging, and survival outcomes. Total ion count normalized of *m*/*z* 2905.0359 released by PNGase F, and distribution of this glycan when cleaved with Endo F3 which remove one core fucose and one core GlcNAc resulting in *m*/*z* 2555.9. The scale bar indicates 2 mm. The heatmap color was generated to show normalized TIC.

Many researchers use the 4T1 mouse mammary tumor cell line in the orthotopic setting to evaluate spontaneous metastasis in the in vivo setting. To determine if any of the core-fucosylated N-glycans that we identified in 4T1 cells were also expressed under the physiological conditions associated with tumors, we orthotopically implanted 4T1 tumor cells into the murine mammary gland and collected tumors for N-glycan analysis. Hematoxylin and eosin (H&E) staining is provided to delineate the tumor shape for comparison to the MALDI-IMS we performed. For this analysis, formalin-fixed paraffin embedded (FFPE) tumor sections were treated sequentially with endoglycosidase F3 (Endo F3) followed with PNGase F to release *N*-glycans and evaluated by MALDI-IMS (Figure 4A). Endo F3 is an endoglycosidase that will cleave within the first and second GlcNAc of the core *N*-glycan with high specificity to *N*-linked glycan containing fucose attached on the first GlcNAc (core fucose). The product of this enzyme will be free N-glycan without the first core GlcNAc (*m*/*z* 203.0794) and fucose (*m*/*z* 146.059). PNGase F is an amidase that will cleave the innermost N-acetylglucosamine (GlcNAc) and asparagine residue on the peptide. The product of this enzymatic reaction is the free *N*-glycan. Structures were then confirmed using HILIC UPLC. By this methodology, we identified three fucose-containing *N*-glycans from the 4T1 tumor tissue that were also identified to be core-fucosylated in the 4T1 cells by HILIC UPLC (Figure 4B). To determine if any of these three *N*-glycans were also found in human breast cancers, we obtained tumor tissue microarrays (TMA) that represented 33 breast cancer patients and included samples from the tumor, normal adjacent tissue, normal lymph node, and tumor containing lymph node. We imaged these tissues after sequential treatment with Endo F3, which cleaves within the fucosylated chitobiose core of asparagine (*N*)-linked bi- and tri-antennary oligosaccharides of glycoprotein [25], and PNGase F, as previously described [26]. Therefore, the comparison of the results from these two enzyme treatments allowed us to evaluate core-fucosylated *N*-glycans in these samples, and confirmed the expression of all three core-fucosylated N-glycans we identified in 4T1 cells, as shown in Figure 4B. Figure 4C shows representative imaging for F(6)A4G4Lac1.

### 2.4. Core-Fucosylated N-Glycans Are Elevated in Lymph Node Metastasis and Tumors from Patients with Recurrence of Disease

**Figure 5:**
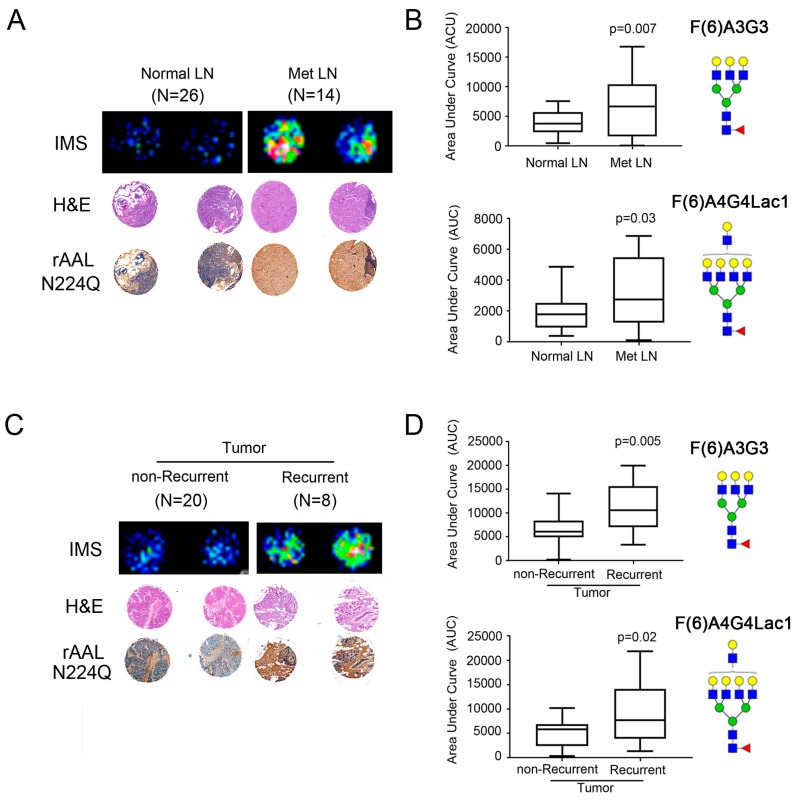
Increased fucosylation in metastatic lymph nodes (met LN) and tumors from recurrent patients. (**A**) A representative comparison of a patient with normal LN and met LN (*n* = 33 patients) showed the increased distribution of fucosylated glycan (F(6)A4G4Lac1 shown here). The H&E stain was used to show the tumor region. Increased core fucosylation in met LN and tumors from patients with recurrence confirmed with lectin staining using rAALN224Q, a lectin with enhanced binding to core-fucosylated structures. TMA cores were imaged at 50× magnification. (**B**) Statistically significant higher core fucosylated tri-antennary glycans (F(6)A3G3) and core fucosylated tetra-antennary with one lactosamine arm (F(6)A4G4Lac1) in the met LN samples (*p* < 0.05). (**C**) A representative comparison of a patient with non-recurrent and recurrent tumors (*n* = 33 patients) showed the increased distribution of fucosylated glycan by MALDI-IMS, with H&E staining, and confirmed with lectin staining using rAALN224Q. TMA cores were imaged at 50× magnification. (**D**) Statistically significant higher core fucosylated tri-antennary glycans (F(6)A3G3) and core fucosylated tetra-antennary with one lactosamine arm (F(6)A4G4Lac1) in the met LN samples (*p* < 0.05).

Using the TMA imaging from the Endo F3 analysis, we evaluated the expression levels of two of the *N*-glycans, (F(6)A3G3 and F(6)A4G4Lac1), we co-identified in the 4T1 and human tumor tissues. From the imaging, we saw that cores from metastatic lymph nodes (met LN; *n* = 14) had high expression of these specific glycans when compared to normal lymph node (normal LN; *n* = 27) samples (Figure 5A; Top-representative cores). We also used a mutant form of recombinant *Aleuria aurantia* lectin N244Q (rAALN224Q), which enhanced binding to core-fucosylated glycans [2,27] to perform IHC on the TMA cores and found higher levels of expression in cores from met LN (Figure 5A and Figure S2). Quantitation of peak values for each glycan mass as area under the curve (AUC) was evaluated for all lymph node TMA samples and we found that compared to normal lymph nodes, metastatic lymph nodes had a ~2-fold higher expression level of core-fucosylated tri-antennary glycan (F(6)A3G3; *p* = 0.007) and core-fucosylated tetra-antennary glycan with a single polylactosamine arm (F(6)A4G4Lac1; *p* = 0.03) (Figure 5B). We also compared tumor tissue samples from patients who experienced a recurrence compared to patients who did not and found that tumors from patients who had recurrent disease had higher levels of both glycans, as well as rAALN224Q staining (Figure 5C and Figure S2), although the latter was not statistically significant. Quantitation of AUC values for F(6)A3G3 and F(6)A4G4Lac1 also showed a ~2-fold higher expression level that was statistically significant for these two N-glycans in tumors from recurrent (*n* = 8) patients compared to non-recurrent (*n* = 20) patients (F(6)A3G3; *p* = 0.005 and F(6)A4G4Lac1; *p* = 0.02) (Figure 5D).

### 2.5. Core-Fucosylated Tetra-Antennary Polylactosamine Is Associated with Poor Survival

**Figure 6:**
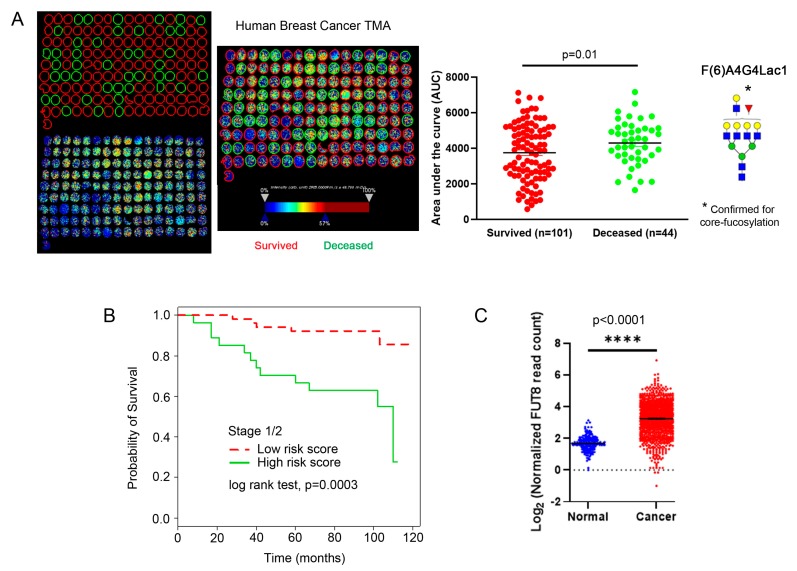
A validation of breast TMA with tumor cores from 145 patients. (**A**) MALDI-IMS of this TMA showing representative deceased (green circles) and survived (red circles) patients from stage 1 and 2 samples. Quantitation of each group shows that tumor samples from deceased patients had statistically significant higher levels of F(6)A4G4Lac1 than those from survived patients (*p* = 0.01). (**B**) Patients with the higher signal of tetra-antennary with one lactosamine arm and one fucose (F(6)A4G4Lac1) correlate with higher risk and poor survival (measured in months). (**C**) Analysis of RNA expression of *FUT8* from TCGA shows higher levels of *FUT8* in cancer vs. normal patients. **** *p* < 0.0001.

To further evaluate the importance of these core-fucosylated N-glycans in breast cancer outcome, we evaluated a second TMA that contained 145 breast tumor cores that included outcome data (i.e., survival data) and pathology diagnosis—i.e., intraductal carcinoma (*n* = 1), invasive lobular carcinoma (*n* = 4); mucinous carcinoma (*n* = 2); invasive ductal carcinoma (*n* = 138). We found that core-fucosylated tetra-antennary glycan containing a single N-acetyllactosamine branch (F(6)A4G4Lac1) was expressed more highly (*p* = 0.01) in tumors from early stage (stage 1 and 2) patients that were deceased compared to those that survived (Figure 6A). We then applied a Cox Proportional Hazard model to predict each patient’s survival risk score and subsequently calculated the mean of risk scores for all patients. When we categorized patients as those with risk scores higher than the mean and those below the mean, we were able to estimate survival functions of these two risk groups with Kaplan-Meier estimator. We found that those with higher expression of core-fucosylated tetra-antennary glycan containing a single *N*-acetyllactosamine (F(6)A4G4Lac1) had an increased likelihood of poor survival outcome compared to those with low expression (Figure 6B). Since these glycans are core-fucosylated and therefore generated through the function of FUT8, which is the only major enzyme required for the addition of core fucose, we also evaluated *FUT8* expression using data mined from The Cancer Genome Atlas (TCGA). We found that *FUT8* expression was significantly elevated in breast cancer vs. normal samples (Figure 6C, *p* < 0.0001), supporting our current findings that core-fucosylation is a driving factor in breast cancer progression.

## 3. Discussion

Here we analyzed the mouse 4T1 metastatic mammary tumor cell line and orthotopic tumors generated from these cells for expression of core-fucose containing *N*-glycans. We identified three individual *N*-glycans, F(6)A2G2, F(6)A3G3, and F(6)A4G4Lac1, that we also identified in human breast cancer tissue microarrays that were analyzed for *N*-glycan expression by MALDI-IMS. Yuan et al. showed that treatment of MDA-MB-231 cells, a human metastatic breast cancer cell line, with α-l-fucosidase (α-l-f) inhibited invasion through the human extracellular matrix (ECM) by transwell invasion assay [15]. Tu et al. showed that treatment of human MDA-MB-231 breast cancer cells or 4T1 mouse mammary tumor cells with 2-fluorinated-peracetyl-fucose, a FUT inhibitor, likewise impaired in vitro cell migration as well as in vivo metastasis using a xenograft assay [23]. Additional experiments from the Tu et al. study showed that knockdown of *FUT8/Fut8* using shRNA also reduced cell migration and metastasis, suggesting that core fucosylation is inherent to this process [23]. Using 2-fluoro-L-fucose (2FF), an easily handled L-fucose analog, that hinders GDP-fucose synthesis, we found that inhibiting fucosylation reduced the migration of 4T1 cells. Our findings confirm and advance previous findings that show the importance of fucose in regulating breast cancer cell migration and invasion [14,23].

*FUT8*, the gene encoding the only enzyme responsible for core-fucose modification was shown to have a higher expression level in highly invasive MDA-MD-231 breast cancer cells [23]. *FUT8* knockdown suppressed both migration and invasiveness and inhibited TGF-β1 signaling [23]. The 4T1 mammary tumor metastasis model was used to evaluate the effect of *Fut8* knockdown in vivo and showed that lung metastasis was reduced with the loss of *Fut8* [23]. However, there was no effect on mammary tumor growth with the knockdown of *Fut8* [23]. Our findings are consistent with these observations as we see reduced 4T1 cell migration and TGF-β1 signaling in the absence of changes in cell proliferation when we treat cells with the l-fucose analog, 2FF. Interestingly, other studies in hepatocellular carcinoma models show slightly different responses to modulation of *FUT8,* where knockdown in HepG2 cells suppressed cell proliferation, migration, and tumor growth [28]. However, it is noted that HL-60, Ramos, and CHO-K1 cells were also tested, and proliferation was not impaired, suggesting organ and/or cell type-specific differences in how fucose is utilized. The findings in HepG2 cells also differ from observations using mammary tumor cells where proliferation is not inhibited, again pointing to cell-specific phenotypes based on fucose status.

The current investigation identified that one product of FUT8, a core-fucosylated N-glycan, F(6)A4G4Lac1, has a correlation with poor clinical outcomes. This result is specific to this glycan, because the other FUT8 product upregulated in metastatic lymph nodes and recurrent tumor; i.e., F(6)A3G3 has no difference with the clinical outcome. Moreover, F(6)A4G4Lac1 and F(6)A3G3 show differential expression levels when evaluating normal adjacent tissue levels compared to expression in tumor tissue. We found that only one of these *N*-glycans, F(6)A3G3, was significantly higher in tumor samples (F(6)A3G2; *p* = 0.004) compared to normal adjacent tissue, whereas the other glycan (F(6)A4G4Lac1) showed no difference in expression between normal adjacent and tumor tissue (Figure S3). Since the data we obtained from MALDI-IMS of breast tumor microarray cores suggests that high expression of F(6)A4G4Lac1 in tumor samples from stage 1 and 2 patients correlates with poor survival, it is possible that these differences are due to the importance of each individual glycan in different stages of cancer progression. It can be proposed that F(6)A3G3 expression is elevated at an earlier stage (i.e., during tumor initiation) compared to F(6)A4G4Lac1, where a high expression in the already developed tumor could signify metastatic progression. Moreover, general analysis of *FUT8* expression using data mined from TCGA showed a significant increase in *FUT8* expression in cancer tissues compared to normal tissues. However, these data are limited by a lack of breast cancer subtype data.

Our findings are supported by two independent studies from Yue et al. and Andres et al. [21,22]. These studies showed *FUT8* was one of the high-expression RNAs isolated from invasive ductal carcinomas (IDC) and was associated with lymph node metastasis, reduced time to disease-related mortality, and recurrence times [22]. The Andres et al. study further suggested that FUT8 expression was associated with a hazard ratio (HR) of 0.82 indicating an 18% deceased of mortality risk for every 2-fold increase of gene expression [22]. Although our study did not measure the expression of *FUT8* directly, by using MALDI-IMS we measured a 2-fold increase of FUT8 products in F(6)A3G3 and F(6)A4G4Lac1. We compared our findings with The Cancer Genome Atlas (TCGA) database and also found ~2-fold change increase in the log_2_ scale of FUT8 RNA in breast cancer samples (*n* = 1076) compared to normal tissues (*n* = 215).

Additionally, our study revealed that F(6)A4G4Lac1 and not F(6)A3G3 was correlated with poor survival. The F(6)A4G4Lac1 is relatively larger than F(6)A3G3 (m/z 2905.0359 and 2174.7715, respectively). The N-glycan size increases in part because of N-acetylglucosaminyltransferase IV (MGAT4) and *N*-acetylglucosaminyltransferase V (MGAT5) activities, which catalyzed the GlcNAc branching. Increased branching may lead to increase in the N-acetyllactosamine (LacNAc) units which in turn enhance lattice formation of poly-LacNAc to prolonged growth factor signaling and generation of outer arm fucosylation sialyl Lewis x, recognized by galectin and selectin, respectively [29]. We observed from human breast tumor TMA of cancer of stages 1 and 2 that F(6)A4G4Lac1 correlated with poor survival and posit the potential of this particular glycan as a breast cancer biomarker.

At least one prior study suggests that polylactosamine (poly-LacNAc) containing *N*-glycans are preferentially found in HER2-positive and triple-negative breast cancers and their metastases [19]. While together, these findings collectively implicate *N*-glycans with poly-LacNAc in breast cancer progression, they lack mechanistic insight. Poly-LacNAc *N*-glycans have been shown to have a role in the immune response by having a suppressive effect on lymphocyte activation and promoting receptor aggregation to block immune response [30,31]. They also can inhibit natural killer cells allowing cancer to evade the immune system [30,31]. Furthermore, in melanoma models, the interaction between poly-LacNAcs and galectin-3 (Gal-3) regulates metastasis [32]. Consequently, additional studies are needed to determine the exact nature of these interactions and their mechanistic roles in driving the metastatic process.

## 4. Materials and Methods

### 4.1. Tissue Culture

4T1 cells were maintained in a humidified 5% CO_2_ incubator at 37 °C and grown in RPMI 1640 media (Corning, Corning, NY, USA) supplemented with 10% heat-inactivated Fetal Bovine Serum (FBS) (Gibco, Gaithersburg, MD, USA), l-Glutamine (Corning), and Penicillin-Streptomycin solution (Corning) unless otherwise specified. Also, 2-deoxy-2-fluoro-l-fucose (2FF) was purchased from Carbosynth Limited (Berkshire, UK) and Synthose Inc. (Ontario, Canada)

### 4.2. Cell-Based Mass Spectrometry Imaging

4T1 cells were profiled by MALDI FT-ICR mass spectrometry (7 Tesla solariX™ Legacy, Bruker Daltonics, Bremen, Germany) as described [33,34]. Transients of 512 kiloword were acquired in broadband positive ion mode over *m/z* 500–5000. Data was collected as an average of 232 spectra per cell treatment (*n* = 3). Each spectrum was a sum total of 300 laser shots rastered over a 30 µm diameter area. High mass accuracy was maintained at 10 ppm during acquisition by lockmass on *N*-glycan peak A2F1G2 (*m/z* 1809.6393). Data were recalibrated post-acquisition using DataAnalysis v 5.0 (Bruker).

### 4.3. Migration Assay

Cells were treated with either DMSO or 100 μM 2FF for 48 h prior to counting and plating. Equal numbers of live cells from each treatment group were plated in the top of the transwell inserts (VWR, Radnor, PA, USA) in serum-free media. Normal growth media was placed in the bottom of the transwell apparatus. After 24 h, the transwell membranes were fixed in 4% paraformaldehyde (PFA), and then stained with crystal violet. Images were captured on a light microscope (Labomed, Los Angeles, CA, USA) using a 20Xobjective (total magnification 200X).

### 4.4. Western Blotting and Lectin Blotting

Cells were lysed in buffer containing 50mM Tris-HCl, pH 7.5, 150mM NaCl_2_, 1mM EDTA, 1% Triton X-100 with HALT protease and phosphatase inhibitor cocktail (Thermo Scientific, Waltham, MA, USA). Western and lectin blot analyses were performed on the Protein Simple FluorChem-R imaging system (San Jose, CA, USA). Antibodies are: Phospho-Smad 1/5 (Cell Signaling, Boston, MA, USA), phospho-Smad 2 (Cell Signaling), phospho-Smad3 (Cell Signaling), Smad 1 (Cell Signaling), Smad 5 (Cell Signaling), Smad 2/3 (Cell Signaling), β−tubulin (Santa Cruz Biotechnology, Dallas, TX, USA). The *Aleuria aurantia* lectin (AAL) was purchased from Vector Labs and the recombinant AALN224Q was produced by the Mehta’s lab.

### 4.5. Lectin Histochemistry

A detailed description of lectin histochemistry was described previously [35]. Briefly, the tissue microarray (TMA) slides were deparaffinized and rehydrated through a series of ethanol and water washes. The endogenous peroxidase was blocked using 3% hydrogen peroxide (Sigma Aldrich; St. Louis, MO, USA), followed by an antigen retrieval step (Dako; Santa Clara, CA, USA). Then the tissues were fixed with 4% formaldehyde and permeabilized with 0.5% IGEPAL CA-630 (Fisher Scientific; Pittsburgh, PA, USA). Before lectin staining, the TMA slides were blocked with serum-free protein block (Dako) followed by 0.5 μg/mL biotinylated recombinant AALN224Q lectin with enhanced binding to core-fucosylated structures [2,27] for 30 min in a humidified chamber. Then, the biotinylated lectin was detected with streptavidin-horseradish peroxidase (Vector Laboratory, Burlingame, CA, USA) and developed with 3.3′-diaminobezidine (DAB) chromogen (Dako). The counterstain was performed with Harris-modified hematoxylin (Fisher Scientific). The image was taken using Hamamatsu Nanozoomer slide scanner, performed by Winship Core Pathology Lab in Emory University, Atlanta, GA, USA, and quantified using the Positive Pixel signal algorithm by Aperio ImageScope, Leica Biosystems Inc, Buffalo Grove, IL, USA.

### 4.6. Proliferation and Colony Formation Assays

For cell counting assays, 100,000 cells per well were plated into 24 well plates. DMSO or 100 μM 2FF was added on one day after plating and refreshed every two days. Cell counting was performed each day for seven days, using a Luna-FL (Logos Biosystems, Annandale, VA, USA) cell counter. Replicates (*n* = 6) were evaluated for each cell type per experiment and each experiment was performed three times.

For colony formation assays, 2,000 cells per well were plated into a 6-well dish. Cells were treated with vehicle (DMSO) or 100 μM 2FF one day after plating. Media and drug were refreshed every three days. On day 14 of the assay, cells were stained with crystal violet, and colonies were quantitated. Replicates (*n* = 3) were evaluated per experiment and each experiment was performed three times.

### 4.7. Exoglycosidase Digestion Coupled to UPLC

Samples were prepared for PNGase F-released glycan analysis. Following PNGase F treatment, samples were labeled with 2AB dye and analyzed using HILIC UPLC (Acquity UPLC System, Waters Corp., Milford, MA, USA). The 2-AB glucose homopolymer standard (Prozyme; Hayward, CA, USA) was used as an internal standard to calculate glucose unit (GU). Following PNGase F treatments, samples were treated with exoglycosidases for glycan sequencing. Exoglycosidases: Sialidase A, almond meal fucosidase, bovine kidney fucosidase, and jack bean mannosidase used in this experiment were purchased from New England BioLab (Ipswich, MA, USA) and ProZyme. Details of the protocol and method of this experiment were described elsewhere [35,36].

### 4.8. Endo F3 and PNGase F Enzymatic Digestion and α-Cyano-4-Hydroxycinnamic Acid (CHCA) Matrix Application

Recombinant Peptide:N Glycanase F (PNGase F) was expressed and purified in the Mehta lab as previously described [34]. Additionally, the Mehta lab also produced recombinant endoglycosidase F3 (Endo F3) in *E. coli.* The detailed process of the tissue preparation, the enzymatic digestion and α-cyano-4-hydroxycinnaminic acid (CHCA) matrix application using the M3 TM-Sprayer™ Tissue MALDI Sample Preparation System (HTX Technologies LLC, Chapel Hill, NC, USA) were performed as described previously [26,34,37,38]. Glycan data with complete structure released from PNGase F and Endo F3 without two dissacharides (GlcNAc and core fucose) were built and checked against GlycoWorkBench database and drawn using GlycoWorkBench as well [39].

### 4.9. MALDI Imaging Mass Spectrometry of N-Glycan Released from Tissues

Mouse tumor tissue and human breast tumor TMAs were analyzed in positive ion mode using MALDI-FT-ICR (solariX Legacy 7.0 Tesla, Bruker Daltonics, Bremen, Germany), with 200 laser shots per pixel of 25 µm diameter raster spot size over *m/z* 495–5000. The spatial resolution of the TMAs was collected at 100 µm step size. Due to the variability of total cores on each TMA slide, different amounts of positions were collected. For TMAs treated with PNGase F (labeled P1, P2, and P3) a total of 83 regions of 13,761 positions, 73 regions of 15,359 positions and 84 regions of 17,774 positions were collected, respectively. For TMAs treated with Endo F3 (labeled E1, E2, and E3) a total of 78 regions of 14,524 positions, 73 regions of 13,865 positions, and 88 regions of 18,234 positions were collected, respectively. PNGase F and Endo F3 treated slides were imported separately to SCiLS Lab software version 2017a (SCiLS Lab, Bruker Daltonics, Bremen, Germany), normalized to total ion current and area under the peak of m/z related *N*-glycans were calculated. All of the images visualized in flexImaging version 4.1 and SCiLS version 2017a (Bruker Daltonics, Bremen, Germany) were normalized to total ion count. Following the MALDI-IMS, the CHCA matrix was removed by complete submersion of tissue slides into 100% ethanol for 5 min. Then the slides were stained using hematoxylin and eosin (H&E). Images with taken using Hamamatsu Nanozoomer, performed by Winship Core Pathology Lab in Emory University, Atlanta, GA, USA.

### 4.10. Animal Studies

Mice were acquired from Jackson Labs and acclimated for at least three days upon arrival. Also, 6–8-week-old female BALB/c mice underwent abdominal mammary gland injection with 100,000 4T1 cells. Five weeks post-injection, tumors were collected, fixed, and processed for paraffin embedding and MALDI-IMS. All studies were approved and performed under the guidelines of the Medical University of South Carolina (MUSC) IACUC under protocol 2018-00486, which was originally approved as VARA 3456 in January 2016.

### 4.11. Tumor Microarrays

Tissue microarray (TMA) slides were either obtained from the Medical University of South Carolina (MUSC, Charleston, SC, USA) Biorepository or purchased from US Biomax (Rockville, MD, USA). The MUSC TMAs contained cores from 33 patients and contained tumor tissue, normal adjacent tissue, normal lymph node, and tumor lymph node (i.e., metastatic lymph node). Samples were annotated for staging and recurrence. The US Biomax TMA (HBreD145Su02) contained cores from 145 patient and only contained tumor tissue. Samples were annotated for staging and survival outcomes.

### 4.12. Statistics

Student’s T-test was applied to graphs in Figure 1, Figure 2, Figure 5 and Figure 6 and represented using the standard error of the mean (± s.e.m.). Survival analysis was performed on all patients and patients grouped within specific stages. Survival of those with late-stage cancer was found to be poor regardless of glycan signature. In the case of those with early-stage breast cancer (stage 1 and 2), a Cox Proportional Hazard model was fitted with glycans. After model selection, an optimized model of four glycans that was statistically associated with survival probability was derived. This Cox Proportional Hazard model was applied to predict each patient’s survival risk score, and subsequently, the mean of risk scores for all patients was calculated. Patients were classified as those with risk scores higher than the mean and those below the mean who were classified as those with a low-risk score. Survival functions of these two risk groups were estimated using Kaplan-Meier estimator. A log-rank test was applied to compare the hazard functions of the two groups. For differential FUT8 RNA expression, normal samples from the Genotype-Tissue Expression (GTEx) project database was compared to breast cancer data from The Cancer Genome Atlas (TCGA) database (http:///cancergenome.nih.gov/). Data are presented as a log2 transformation and Student’s t-test were performed.

## 5. Conclusions

The current study examined core-fucosylation in breast cancer progression. Findings provide further evidence that impairing core-fucosylation inhibits the metastatic potential of breast cancer cells. Importantly, the data support a role for core-fucosylated *N*-glycans in breast cancer at an early stage prior to metastasis. The data further depicts that a core-fucose tetra-antennary *N*-glycan containing a single *N*-acetyllactosamine arm, F(6)A4G4Lac1, is correlated with poor clinical outcomes in breast cancer. Taken together, our study indicates that core-fucose modification of N-glycans may be a potential therapeutic target and early stage marker depicting poor prognosis.

## Supplementary Materials

Supplementary materials can be found at https://www.mdpi.com/1422-0067/20/10/2528/s1.

## Abbreviations

TMA	Tissue Microarray
H&E	hematoxylin and eosin
CHCA	α-cyano-4-hydroxycinnamic acid
Endo F3	endoglycosidase F3
PNGase F	Peptide: N-glycosidase F
AAL	*Aleuria aurantia* lectin
LacNAc	*N*-acetyllactosamine
AUC	Area under curve
SA	Sialidase A
NeuAc	*N*-acetyl neuraminic acid
NeuGc	*N*-glycolylneuraminic acid
AMF	Almond meal fucosidase
BKF	Bovine kidney fucosidase
FFPE	Formalin-fixed paraffin embedded
