# Supplementary material for: Core-Fucosylated Tetra-Antennary N-Glycan Containing A Single N-Acetyllactosamine Branch Is Associated with Poor Survival Outcome in Breast Cancer
Source: Int J Mol Sci. 2019 May 23;20(10):2528. doi: 10.3390/ijms20102528 (PMC6566954; doi:10.3390/ijms20102528)
Supplement: Supplementary file 1 [file ijms-20-02528-s001.pdf]

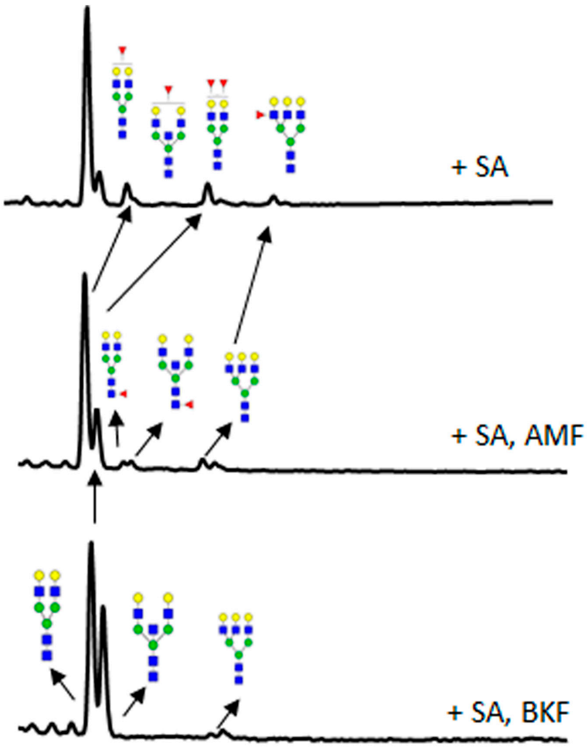

Supplemental Figure 1. Ceruloplasmin protein control for testing α1-2,3,4- fucosidase (outer arm) almond meal fucosidase (AMF) and all fucose linkages including α1,6-fucose (core) bovine kidney fucosidase (BKF), to show AMF cleaved 7% of outer arm fucose and BKF cleaved remaining 14% core fucose on this standard protein.

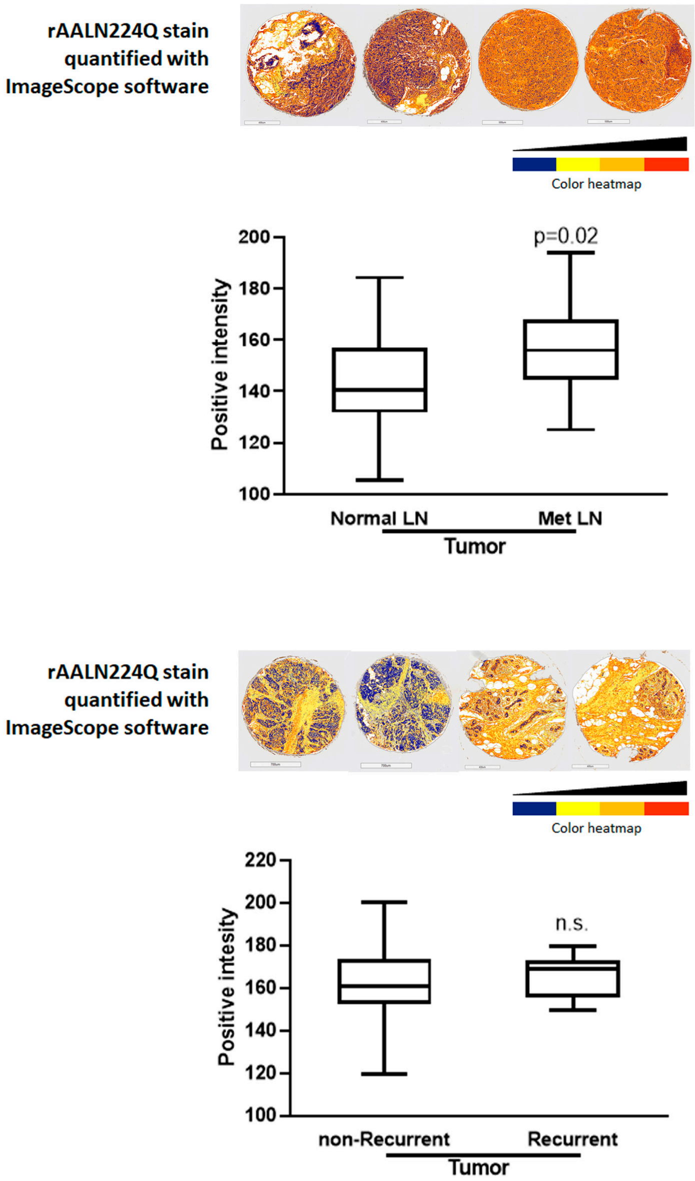

Supplemental Figure 2. Quantitation of positive rAALN224Q staining from the TMA shown in Figures 4C and 5.

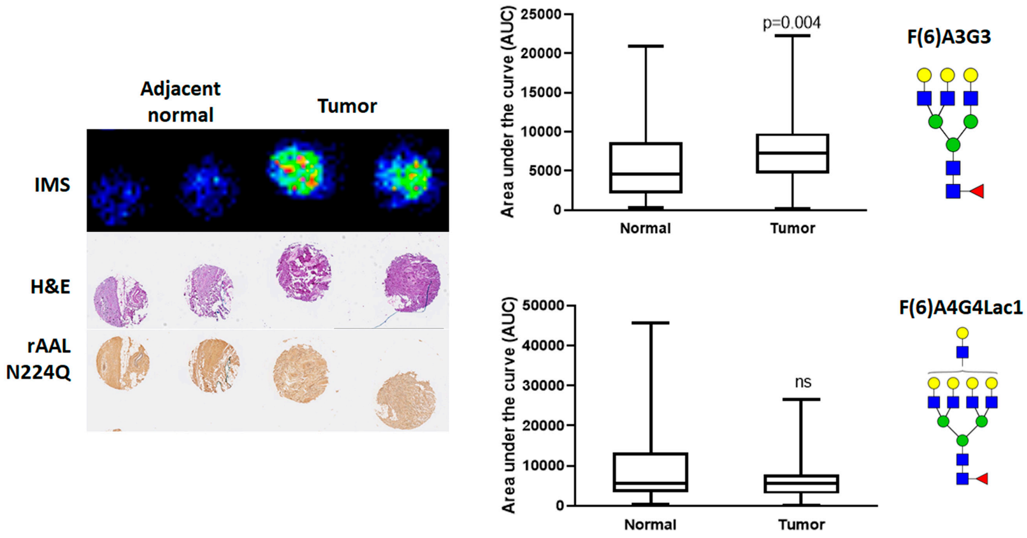

Supplemental Figure 3. Quantification of F(6)A3G3 and F(6)A4G4Lac1 in adjacent normal tissue compared to tumor tissue from the TMA shown in Figures 4C and 5.
